# Ring Chromosome 4 in a Child with Multiple Congenital Abnormalities: A Case Report and Review of the Literature

**DOI:** 10.1155/2016/4645716

**Published:** 2016-08-16

**Authors:** C. S. Paththinige, N. D. Sirisena, U. G. I. U. Kariyawasam, L. P. C. Saman Kumara, V. H. W. Dissanayake

**Affiliations:** ^1^Human Genetics Unit, Faculty of Medicine, University of Colombo, 00800 Colombo, Sri Lanka; ^2^Castle Street Hospital for Women, 00800 Colombo, Sri Lanka

## Abstract

A female child born preterm with intrauterine growth retardation and presenting with facial dysmorphism with clefts, microcephaly, limb deformities, and congenital abnormalities involving cardiovascular and urinary systems is described. Chromosomal analysis showed a* de novo* 46,XX,r(4)(p15.3q35) karyotype. The clinical features of the patient were compared with the phenotypic characteristics of 17 previously reported cases with ring chromosome 4 and those with Wolf-Hirschhorn syndrome (4p-). Clinical features observed in this case are consistent with the consensus phenotype in ring chromosome 4. Patent ductus arteriosus and bilateral talipes equinovarus observed in this baby widen the phenotypic spectrum associated with ring chromosome 4.

## 1. Introduction

Ring chromosome is a rare form of structural chromosomal abnormality which commonly results from the breakage of an end segment of both the short and long arms of the chromosome and subsequent end joining. The site of breakage and the amount of chromosomal material lost vary from case to case even when a single chromosome is considered. The cytogenetic variation in the presence of a ring chromosome depends on the ring size, rate of sister chromatid exchange events, and the viability of altered cell lines [1, 2]. Phenotypic variation of these individuals depends on the size of the ring chromosome, amount of genetic material lost in breakage, the stability of the ring chromosome, and the presence of secondary chromosomal aberrations including the varying degrees of mosaicism [1]. Subtelomeric or telomere-to-telomere fusion of the chromosomes resulting in formation of complete rings was also reported, usually with milder phenotypic changes due to the minimal loss of genetic material [3–5]. Advances in the cytogenetic techniques, such as high resolution molecular karyotyping, have allowed the detection of novel mechanisms of ring formation, for example, in patients with inverted duplication and terminal deletion, where ring formation was observed as an escape mechanism [6].

Ring chromosomes account for a very low percentage of structural chromosomal abnormalities [7, 8] and majority of the cases are sporadic arising* de novo* [9]. Ring formation is reported in all human chromosomes with nearly 50% of rings arising from acrocentric chromosomes. Among the nonacrocentric human autosomes, ring chromosome 4 was observed to be a relatively commoner occurrence [8], but only about 20 cases have so far been reported in detail.

The first report of ring chromosome 4 was in 1969, in a newborn baby with growth retardation and multiple congenital abnormalities affecting many systems resulting in early neonatal death [10]. The most recent review of sixteen cases with ring chromosome 4, in 2006, highlighted low birth weight, growth retardation with retarded bone age, microcephaly, and mental retardation as the main clinical features observed in majority of cases. Cleft lip and cleft palate, abnormal facial features, and skeletal abnormalities of the hands and feet were also observed commonly while involvement of one or more of the cardiovascular, gastrointestinal, and genitourinary systems was observed in some cases [11]. It is suggested that the phenotype observed in ring chromosome 4 is a combination of clinical features due to terminal deletions of the chromosome and unspecified developmental abnormalities due to chromosomal instability in ring formation [12].

We report a baby girl who was referred for genetic evaluation due to intrauterine growth retardation and multiple congenital abnormalities and was found to have a* de novo* 46,XX,r(4)(p15.3q35) karyotype. Herein, her clinical findings are compared with those of the previously reported cases of ring chromosome 4.

## 2. Case Description

Clinical information and peripheral blood samples were collected from the proband and his parents after obtaining their written informed consent. Parental written informed consent was also obtained for publication.

The proband was the first child of a nonconsanguineous couple with an unremarkable family history. At the time of conception, the father was 39 years old while the mother was 33 years old. Routine prenatal scans indicated intrauterine growth retardation. The baby was delivered at 35 weeks' gestation. She was resuscitated at birth and Apgar scores were 4, 6, and 6 at 1 minute, 5 minutes, and 10 minutes, respectively. Her birth weight (1.5 kg) and head circumference (25.5 cm) were below the 3rd centile expected for a baby born at 35 weeks of gestation and the crown-to-heel length (48 cm) was above the 50th centile. The baby had microcephaly, right side ptosis, low set ears, unilateral cleft lip and cleft palate, short neck, and bilateral talipes equinovarus. A murmur was elicited during the cardiovascular examination. Examination of other systems showed no abnormality.

Ductus arteriosus was found to be patent in echocardiography performed at 3 weeks and 9 weeks after birth. Abdominal ultrasonography revealed renal agenesis in the right side. No abnormalities were detected in the ultrasound scan of the brain. Radiological assessment of the chest and spine was normal.

**Figure 1:**
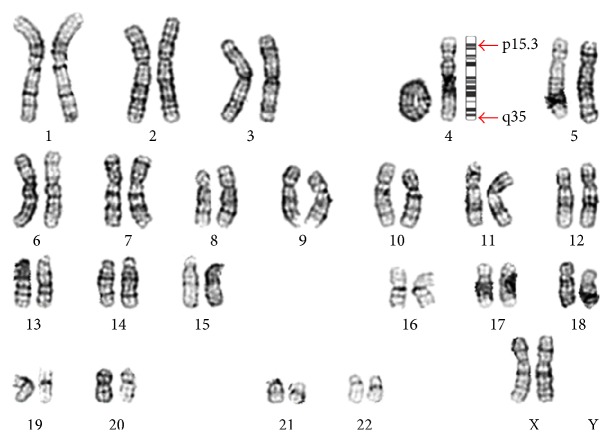
Karyogram of the baby showing ring chromosome 4.

Chromosomal analysis of the proband's peripheral blood using the GTL banding technique at 525-band resolution showed ring chromosome 4 in all 20 spreads analyzed (Figure 1). The karyotype was 46,XX,r(4)(p15.3q35). Chromosomal analysis of both the mother and father was normal.

The baby died of respiratory distress resulting from cardiac failure and renal impairment at 10 weeks after birth. Autopsy was not done.

## 3. Discussion

Since the first report in 1969, over 20 cases have so far been reported describing the phenotypic spectrum associated with ring chromosome 4. The clinical signs of the present case in comparison with the phenotypic findings of 17 previously reported cases [3, 4, 10–24] are summarized in Table 1. Almost all the patients reported had both prenatal and postnatal growth retardation and microcephaly. In addition, most of them also had developmental delay and mental retardation. Abnormal facial features, which were commonly observed in these patients, included hypertelorism, epicanthal folds, micrognathia, and abnormalities of the nose and ears. Five of the 16 reported patients had cleft lip and/or cleft palate. Skeletal abnormalities were also a common finding among these patients. These abnormalities commonly involved the thumb, fifth finger, and feet. Abnormalities of feet reported among these patients include overlapping toes, hypoplasia of toes, valgus deformity, and rocker-bottom feet. In addition to these phenotypic features observed in ring chromosome 4, this baby had bilateral talipes equinovarus. Abnormalities of the heart, gastrointestinal system, kidneys, and urinary system were observed in nearly one-third of the reported patients. As reported in 4 patients, these systemic abnormalities tended to occur together [10, 11, 19, 23]. Cardiac abnormalities observed in patients with ring chromosome 4 typically involve cardiac septation and include atrial septal defects, patent foramen ovale, ventricular septal defects, and transposition of great arteries. Patent ductus arteriosus observed in this baby is not reported earlier in association with ring chromosome 4. Unilateral renal agenesis observed in this baby is also reported in one case previously, while renal hypoplasia is observed in 3 other patients. These clinical features observed in this baby are consistent with the consensus phenotype of ring chromosome 4; furthermore bilateral talipes equinovarus and patent ductus arteriosus add to the phenotypic spectrum observed in ring chromosome 4.

In the reported cases of ring chromosome 4, chromosomal breakpoints were common at p16 and q35. Telomere-to-telomere fusion was suggested as the mechanism of ring formation in 2 cases, and both these cases did not show any systemic involvement [3, 4]. Mosaic genotype with ring chromosome 4 was also reported in 2 cases. One case with 46X,r(4)/46,XY mosaicism had significant renal involvement [20], while the other one had 45,XX,-4/46,XX,r(4) mosaicism which was detected prenatally and the pregnancy was terminated at 17 weeks' gestation. Abnormalities of the cardiovascular and gastrointestinal systems were identified in the fetus [23].

It is postulated that the clinical features observed in patients with ring chromosome 4 are partly due to the terminal deletion of the chromosome's p and q arms. Patients with terminal deletions of the p arm were reported to have growth failure, developmental delay, congenital cardiac defects, finger and toe anomalies, and speech delay [26]. The genes deleted in this critical region in chromosome 4 in those patients included* HAND2* gene, which is expressed in the ventricular chambers of the developing heart, and* SORBS2* gene, which is expressed in epithelial tissue and cardiac muscle. These genes may potentially contribute to the cardiac defects in terminal 4q deletion, hence in ring chromosome 4.

Terminal deletion of the short (p) arm of chromosome 4 is a well-known clinical entity which is described as Wolf-Hirschhorn syndrome (WHS) (MIM 194190). Terminal 4p16.3 deletion is known to be necessary and sufficient to produce the classical phenotype of WHS [25]. A recent review of reported cases of WHS suggested that the characteristic facies, mental retardation, prenatal and postnatal growth retardation, microcephaly, seizures, and hypotonia were present in nearly all the reported cases irrespective of the size of the deleted chromosomal segment. Congenital heart defects, cleft palate and cleft lip, colobomas, hypospadias, and renal and skeletal abnormalities are the other commonly observed abnormalities in WHS [25]. The breakpoint on the short arm is reported to be located in the region 4p16.1 [23]. This leads to deletion of the distal portion of the short arm that includes the WHS critical region (WHSCR). Genotype-phenotype analysis of WHS has reported that haploinsufficiency of* WHSC1* gene causes the characteristic facial dysmorphism and growth delay in WHS. Microcephaly is mapped to an area with its distal boundary at 2.2 Mb from the telomere, whereas the chromosomal localization of cardiac, renal, and genital anomalies is known to be further distal to the telomere [25]. This explains the more severe phenotype with systemic involvement in larger terminal deletion in WHS as well as in ring chromosome 4. The breakpoint in p arm of chromosome 4 is located proximally to* MSX1* gene which is known to be associated with cleft lip and cleft palate [27].

Phenotypic features observed in the present case are comparable to the phenotype of the previously reported cases with r(4)(p16q35) karyotype and some of the phenotypic features observed in 4q terminal deletion and WHS. This suggests that the phenotypic features of ring chromosome 4 are the cumulative effect of the deletion of a number of genes located in the terminal 4p and 4q regions. Chromosomal instability in ring formation is also likely to have an additional influence on the phenotype in ring chromosome 4.

In the literature cited in this paper, 5 of the 17 reviewed reports on ring chromosome 4 have presented their case as WHS [11, 12, 15, 16, 24].  Table 2 shows the clinical features of WHS which were found in common in the present case and in previously reported cases of ring chromosome 4. Although dysmorphic facial features were observed in majority of the cases, the characteristic “Greek warrior helmet” profile with prominent glabella was observed in only two cases [11, 23]. Even though seizures and hypotonia are described as customary features of WHS, seizures were reported in only two cases [11, 13] and hypotonia was reported in another two cases with ring chromosome 4 [15, 16]. These findings suggest that the phenotypic spectrum of patients with ring chromosome 4 does not always conform to the consensus phenotype of WHS.

In conclusion, the phenotypic features observed in this baby add to the spectrum of clinical features observed in ring chromosome 4. Conventional karyotyping was used for cytogenetic diagnosis of the present case; however, the exact chromosomal breakpoints can only be confirmed by the use of molecular cytogenetic methods such as fluorescence* in situ* hybridization (FISH) and microarray, which have not yet been fully established at our centre. Such techniques will allow the precise identification of the deleted chromosomal segments and the genes involved, which will help to improve the understanding of the phenotype-genotype correlation of this relatively rare structural chromosomal abnormality.

## Figures and Tables

**Table 1:** Karyotype and the clinical features of the present case and previously reported cases with ring chromosome 4.

	Present case	1 [10]	2 [13]	3 [14]	4 [3]	5 [15]	6 [16]	7 [17]	8 [18]

Karyotype	46,XX,r(4)	46,XY,r(4)	46,XY,r(4)	46,XY,r(4)	46,XY,r(4),(p16q35)	46,XY,r(4),(p15q35)	46,XX,r(4),(p16q35)	46,XY,r(4),(p16q35)	46,XY,r(4),(p16q35)
Preterm delivery	+								+
Low birth weight	+	+	+	+	+	+	+	+	+
Growth retardation	+	+	+	+	+	+	+	+	+
Developmental delay	NA			+	+			+	+
Mental retardation	NA	NA	+	+	+	NA	NA		+
Microcephaly	+	+	+	+	+		+	+	+
Hypertelorism	−		+			+	+		
Epicanthal folds	−		+			+			
Coloboma	−	+				+			
Ptosis	+			+					
Malformed ears	Low set	Large	Low set			Low set	+	+	
Abnormal nose	−	Flat, broad					Flat	Broad	
Cleft lip	+ (U/L)	+ (U/L)				+ (B/L)			
Cleft palate	+	+				+		+	
Micrognathia	−		+					+	+
Abnormal spine	−								Mild kyphosis
Transverse palmar crease	−	+	+					+	
Clinodactyly	−		+	+	+	+			
Abnormal thumb	−	B/L hypoplasia		Hypoplasia	Long slender				Adducted
Feet deformity	+ B/L TEV	Overriding toes			Valgus deformity	Rocker-bottom, overriding toes	Valgus deformity	Overlapping toes	Syndactyly, hypoplastic toes
Sacral dimple	−		+		+				
Cardiac abnormalities	PDA	PFO					VSD		
Intestinal anomalies	−	Incomplete rotation							
Renal and urinary tract anomalies	Unilateral agenesis	Renal hypoplasia							
Hypospadias	NA	+	Epispadias	+			NA	+	
Cryptorchidism	NA		+	+			NA		+
Neurological abnormalities	−	Generalized hypoplasia of brain	Seizures			Hypotonia	Hypotonia abductors		
Early death	10 weeks	4 weeks				2nd week	3 days		

	9 [12]	10 [19]	11 [4]	12 [20]	13 [21]	14 [22]	15 [23]	16 [11]	17 [24]

Karyotype	46,XX,r(4),(p16q35)	46,XX,r(4),(p16q35)	46,XY,r(4)	46,XY,r(4)(p16q35)/46,XY	46,XY,r(4),(p16q35)	46,XX,r(4),(p16.3q35.2)	45,XX,-4/46,XX,r(4)	46,XY,r(4),(p16.3q35)	46,XX,r(4)
Preterm delivery		+					Elective termination	+	
Low birth weight	+	+	+	+	+		NA	+	+
Growth retardation	+	+	+	+	+	+	NA	+	+
Developmental delay	+	+		+	+		NA	+	
Mental retardation	+	+		+			NA	+	
Microcephaly	+	+	+	+	+	+		+	+
Hypertelorism					+		+	+	
Epicanthal folds		+		+	+	+			
Coloboma								+	
Ptosis								+	
Malformed ears	Large	+		+				Large, flat	Posteriorly rotated
Abnormal nose		Depressed bridge		Beaked nose	Short, bulbous tip		High bridge		
Cleft lip							+(B/L)	+	
Cleft palate							+	+	
Micrognathia		+		+	+				+
Abnormal spine				Mild kyphoscoliosis					
Transverse palmar crease						+			
Clinodactyly			+	+	+				
Abnormal thumb					Proximally placed			B/L hypoplasia	
Feet deformity									
Sacral dimple		+							
Cardiac abnormalities	ASD	VSD					Dextrocardia/ TGA	IVS aneurysmal dilation	
Intestinal anomalies		Duodenal atresia					Gallbladder hypoplasia	Midgut malrotation	
Renal and urinary tract anomalies		VUR		Oligomeganephronia	Hypoplastic ectopic kidney			Hypoplasia	Unilateral agenesis
Hypospadias	NA	NA				NA	NA	+	NA
Cryptorchidism	NA	NA		+		NA	NA	+	NA
Neurological abnormalities								Seizures Cerebral atrophy	
Early death							NA		

NA: not applicable, U/L: unilateral, B/L: bilateral, PDA: patent ductus arteriosus, ASD: atrial septal defect, VSD: ventricular septal defect, TGA: transposition of great arteries, and IVS: interventricular septum.

**Table 2:** The comparison of clinical features of Wolf-Hirschhorn syndrome which were found in common in the present case and in previously reported cases of ring chromosome 4.

Clinical signs associated with WHS (Zollino et al., 2008 [25])	Number of reported cases of ring chromosome 4	Present case
Facial dysmorphism (2 or more abnormal facial features, excluding facial clefts)	11/17	+
Mental retardation	8/13	NA
Seizures	2/16	−
Prenatal growth retardation	15/16	+
Postnatal growth retardation	16/16	+
Microcephaly	15/16	+
Hypotonia	2/16	−
Congenital heart defects	6/17	+
Cleft lip/cleft palate	5/17	+
Ocular colobomas	3/17	−
Hypospadias	4/11	NA
Renal abnormalities	6/17	+
Skeletal abnormalities	11/17	+

WHS: Wolf-Hirschhorn syndrome. NA: not applicable.
